# Soliton solutions of fractional extended nonlinear Schrödinger equation arising in plasma physics and nonlinear optical fiber

**DOI:** 10.1038/s41598-023-37757-y

**Published:** 2023-07-05

**Authors:** Jamshad Ahmad, Sonia Akram, Kanza Noor, Muhammad Nadeem, Amelia Bucur, Yahya Alsayaad

**Affiliations:** 1 Department of Mathematics, Faculty of Science, University of Gujrat, Gujrat, 50700 Pakistan; 2 School of Mathematics and Statistics, Qujing Normal University, Qujing, 655011 China; 3 Department of Mathematics and Informatics, Faculty of Sciences, “Lucian Blaga” University of Sibiu, 550012 Sibiu, Romania; 4 Department of Physics and Mathematics, Zabid-Hodeidah, Hodeidah University, Al-Hudaydah, 4113 Yemen

**Keywords:** Applied mathematics, Fluid dynamics

## Abstract

In this research, we study traveling wave solutions to the fractional extended nonlinear SchrÖdinger equation (NLSE), and the effects of the third-order dispersion parameter. This equation is used to simulate the propagation of femtosecond, plasma physic and in nonlinear optical fiber. To accomplish this goal, we use the extended simple equation approach and the improved *F*-expansion method to secure a variety of distinct solutions in the form of dark, singular, periodic, rational, and exponential waves. Also, the stability of the outcomes is effectively examined. Several graphs have been sketched under appropriate parametric values to reinforce some reported findings. Computational work along with a graphical demonstration confirms the exactness of the proposed methods. The issue has not previously been investigated by taking into account the impact of the third order dispersion parameter. The main objective of this study is to obtain the different kinds of traveling wave solutions of fractional extended NLSE which are absent in the literature which justify the novelty of this study. We believe that these novel solutions hold a prominent place in the fields of nonlinear sciences and optical engineering because these solutions will enables a through understanding of the development and dynamic nature of such models. The obtained results indicate the reliability, efficiency, and capability of the implemented technique to determine wide-spectral stable traveling wave solutions to nonlinear equations emerging in various branches of scientific, technological, and engineering domains.

## Introduction

Non-linear partial differential equations (NLPDEs) have piqued the interest of scientists in recent years due to their ability to simulate a wide range of captivating non-linear events. They are crucial building blocks for understanding nonlinear processes. NLPDEs have a broad number of applications in scientific disciplines, including fluid dynamics, lattice dynamics, food supplements, nonlinear optical fibres, plasma physics, meteorology, hydrodynamics, medicine, and biology^1–11^. A subfield of physics called fluid mechanics studies the motion of fluids like liquids, gases, and plasmas. Numerous disciplines, such as civil, chemical, engineering, geophysics, oceanography, astronomy, meteorology, and fluid mechanics^12–15^. The most useful generalizations and extensions are normally integrals of integer order and fractional calculus derivatives. The application of fractional order derivatives and integral-based models in particular sectors of science and engineering has been extensively researched by a large number of medical professionals and mathematicians^16–19^. Over the past two centuries, various academics have developed an interest in fractional calculus (FC). They are used to model a variety of nonlinear nature such as, biological and chemical processes, fluid mechanics, etc. Because fractional derivatives are used to better illustrate dynamical systems, Partial differential equations (PDEs) in fractional order are a generalization of PDEs in conventional integer order.

The perturbed fractional NLS equation commonly describes optical fibre communications. Because of the low transverse section of light that exists in an optical fiber, even moderate optical powers result in high optical intensities. This is especially true if brief pulses are transmitted through fibres. In addition, the Kerr effect is the fiber’s most basic and typical nonlinear effect. Moreover, when optical intensity rises, the phase delay in the fiber increases. The Kerr law nonlinearity is revealed by a medium whose refractive index determines the intensity of light travelling through it. The components of dispersion and temporal evolution are aspects of the NLS equation. The fragile balance verifies the stable transmission and presence of such solitons. Additionally, plasmas, which are thought to be the most prevalent type of ordinary stuff in the universe, have been found to be linked to stars. In literature, there are numerous theoretical components to constructing supplementary techniques such as Laskin^20^ use the fractional Hamilton operator and the descriptions of the relationships between the fractional and standard SchrÖdinger equations. Veeresha et al.^21^ investigate the fractional model of Klein–Gordon–SchrÖdinger system with the aid of the q-homotopy analysis method using the Laplace transform. Akinyemi et al.^22^ studied a model of fifth-order weakly non local Schrodinger problem.

A travelling wave is a wave that advances in a particular direction, with the addition of retaining a fixed shape. Moreover, a travelling wave is associated to having a constant velocity throughout its course of propagation. Such waves are observed in many areas of science. To find the explicit solutions to nonlinear problems are of fundamental importance. The travelling wave solutions may be useful in the theoretical and numerical studies of the model systems. Therefore, finding travelling wave solutions of nonlinear equations is of fundamental interest to understand the equations fully. The study of traveling wave solutions of NLPDEs plays an important role to look into the internal mechanism of complicated physical phenomena. In addition, these methodologies’ results lack any analytically expressions. Conversely, analytical approximation techniques are preferred by scientists because they have deeper physical roots and are more deserving of parametric investigation. As a result, many researchers developed various methodologies, including the modified extended direct algebraic scheme^23^, Hirota Bilinear strategy^24^, Elzaki transform^25^, simple equation approach^26^, *F*-expansion scheme^27^, the extended Fan sub-equation strategy^28^, Natural Transform^29^, $sec-csc,~ sech-csch,~ \tan -\cot ,~ \text{ and }~ \tanh -\coth$^30^, $(\frac{G}{G'})$-expansion approach^31^, Darboux transformation scheme^32^, inverse scattering approach^33^, sardar equation method^34^, $\sinh$-Gorden equation approach^35^, and some others^36–43^.

In the scenario when the pulse propagates in a multisolitons regime, the NLSE describing the propagation of picosecond solitons in single-mode optical fibres predicts a periodic change in the shape of the wave packet envelope^44^. The transmission of detectable femtosecond pulses in single-mode optical fibres is fundamentally hampered by nonlinear effects, which cause substantial distortion even at extremely short propagation distances (a few metres). A method for transmitting nanojoule-energy in 100-fs pulses at 800 nm over a short distance of common optical fibre is put forth by Clark^45^. The desired pulses are produced at the fiber’s end by compressing the mode-locked laser’s pulses first spectrally and then temporally. Additionally, for pulse widths of several picoseconds or longer, a description of solitons in optical fibres in terms of the nonlinear Schrodinger equation appears to fit the experimental results well.

Although a number of theoretical articles on the development of the envelope equation have been published, to our knowledge, there is no work that consistently incorporates effects of transverse inhomogeneity and nonlinear dispersion and dissipation at higher orders. Current studies must take into account the effects of the higher orders in order to fully understand the high-power and ultrashort (in the femtosecond regime) pulse propagation experiments seen in the soliton compression and frequency shift^46,47^. In this study, the nonlinear Schrodinger equation’s envelope equation is derived to a high degree, with the material dispersion, radial mode-function profile, and index of refraction profile explicitly providing the coefficients. It is demonstrated that the higher-order dissipation lowers the soliton’s carrier frequency in proportion to the soliton’s amplitude and to the fourth power of the soliton’s propagation distance. In particular, those studies have discussed the possible effects of higher-order dispersive and nonlinear terms.

Among those approaches, the proposed improved *F*-expansion method and the extended simple equation method are reliable and credible mechanisms to construct more general soliton solutions of NLPDEs in engineering and applied sciences. The main benefit of the improved *F*-expansion method and the extended simple equation method over the existing other methods mentioned^48–50^ is that this scheme provide more abundant exact soliton solutions including some novel solutions with additional parameters in a simple and straight way. The exact soliton solutions have its great importance to know entirely the effect of the parameters in any circumstances. To fill the gap of the previous findings, we are motivated to find soliton solutions to the considered model via recent methods known as the extended simple equation approach and the improved *F*-expansion function method. The soliton solutions for extended NLSE play a significant role in soliton theory, physical sciences and optical engineering. So the finding of exact soliton solutions for extended NLSE is very important for mathematicians and physicists^51–55^. To the best of our knowledge, such an abundance of solutions has never been established in earlier literature. The goal of the current research is to determine how to use the extended simple equation approach^56^ and the improved *F*-expansion function method^57^ to acquire the soliton solutions to the studied model. The robustness of the proposed methods is that they provide more abundant exact soliton solutions including some novel solutions with additional parameters in asimple and striaght way. Additionally,the stability property of the obtained soliton wave solutions is discussed based on the Hamiltonian system’s characterizations^58^. It is also noted that the proposed methods are easy to handle and offer a wide range of analytical solutions, which could provide new forms of soliton solutions with a direct application in the optical fiber. The SchrÖdinger equation class is used to model a variety of phenomena, particularly those related to quantum physics, energy, and energy quantization. The nonlinear SchrÖdinger equation is the most typical equation for the development of slowly changing quasi-monochrome wave packets in weak nonlinear media with dispersion, whereas the linear SchrÖdinger equation represents the time evolution of the wave function^59^. It simulates a number of nonlinearity events in fiber, including stimulated Raman scattering, optical solitons, self-phase modulation, second harmonic production, ultrashort pulses, and others. In order to understand fractional quantum mechanics, Laskin proposed the fractional SchrÖdinger equation^60^.

The femtosecond pulse propagation in monomode optical fibre is expressed by the fractional extended NLSE^61–64^ as follows:

1 $$\begin{aligned} i(D^\sigma _x v + \gamma _1 D^\delta _t v) - \frac{\gamma _2}{2} D^{2\delta } _t v + b |v|^2 v + i \psi _1 D^\delta _t (|v|^2 v) + i \psi _2 D^\delta _t (|v|^2) v-i \frac{\gamma _3}{6} D^{3\delta } _t v = 0, \end{aligned}$$

where $v = v(x, t)$ denotes the electric field, $\gamma _1$ shows the inverse of speed, $\gamma _2$ represents the second-order dispersion unknown, $\gamma _3$ denotes the third-order dispersion variable (TOD), $\psi _1$ is the coefficient of the derivative cubic term, $\psi _2$ is for soliton self-frequency shift and $\beta$ is the effective nonlinear coefficient. In this study, we apply the extended simple equation approach and the improved *F*-expansion method to obtain the wave solutions for Eq. (1). The strength of the entire optical framework is determined by the TOD parameter and well-known effects, which also serve as conditional constraints. Securing results for this model that control these occurrences is crucial for a thorough understanding of these physical processes.

The remainder of the paper is summarized after the introduction section. In “Conformable fractional derivative”, we discuss conformable fractional derivative. The improved *F*-expansion scheme and extended simple equation method are discussed in “Algorithm of the methods” of this article. In “Extraction of wave solutions”, we use the aforementioned techniques to extract the periodic type, dark type, singular, and rational soliton solutions of the Eq .(1). In “Stability analysis”, we discuss the analysis of solutions. Graphical representations and results of a few of the generated results are explained in “Results and discussions”. Lastly, we present conclusion in “Conclusion”.

## Conformable fractional derivative

Assume that $D^\sigma _\eta$ is a differential operator of any order, such as $0 < \alpha \le 1$. Then conformable fractional derivative of $V(\eta )$ is given by

2 $$\begin{aligned} D^\sigma _\eta (v(\eta ))=\lim \limits _{\epsilon \rightarrow 0}\frac{v(\eta +\epsilon \eta ^{1-\sigma })-v(\eta )}{\epsilon },~\eta >0. \end{aligned}$$

Following are some characteristics of this definition:

### Theorem 1

*Suppose that function* $v(\eta )$ *and* $w(\eta )$ *are* $\sigma -$*differentiable at* $\eta >0$ *with* $\sigma \in (0,1]$, *therefore*

$$\begin{aligned}{} & {} (i)~ D^\sigma _\eta (\eta ^n)=n\eta ^{n-\sigma }~\forall ~ n\in R.\\{} & {} (ii)~ D^\sigma _\eta (c)=0,~where~ c ~is~ constant.\\{} & {} (iii) ~ D^\sigma _\eta (dv(\eta )+ew(\eta ))= d D^\sigma _\eta (v(\eta )) +eD^\sigma _\eta (w(\eta ))~ \forall ~ d,~e \in R.\\{} & {} (iv)~ D^\sigma _\eta (v(\eta )w(\eta ))=v(\eta )D^\sigma _\eta (v(\eta ))+v(\eta )D^\sigma _\eta (w(\eta )).\\{} & {} (v) ~ D^\sigma _\eta (\frac{v(\eta )}{w(\eta )})=\frac{w(\eta )D^\sigma _\eta (v(\eta ))-v(\eta )D^\sigma _\eta (w(\eta ))}{w^2(\eta )}.\\{} & {} (vi)~ if~ v ~is~ differentiable,~ then~ D^\sigma _\eta (v)(\eta )=\eta ^{1-\sigma }~\frac{dv(\eta )}{d\eta }. \end{aligned}$$

### Theorem 2

*Assume that* $v(\eta )$ *is both differentiable and sigma-differentiable in the range* $\sigma \in (0, 1]$. *Furthermore, let* $v(\eta )$ *be a differentiable function with the same range* $v(\eta )$,

$$\begin{aligned} D^\sigma _\eta (v(\eta ).~w(\eta ))=\eta ^{1-\sigma }v'(\eta )~w'(v(\eta )). \end{aligned}$$

## Algorithm of the methods

The fractional order nonlinear equation with the spatial variable x and time variable t is as follows:

3 $$\begin{aligned} J(v,D^ \delta _t v,D^ {\sigma } _x v,D^ {2\delta } _{tt}v,D^ {2\sigma } _{xx}v, D^\delta _t D^\sigma _x v,\cdots )=0,~0< \delta ,~\sigma \le 1, \end{aligned}$$

where *v*(*x*, *t*) represents the unknown function , and *J* is a polynomial with fractional partial derivatives in *v*(*x*, *t*). The wave variable should be changed as follows:

4 $$\begin{aligned} v(x,t)=V(\chi ) e^{(i \phi )},~\chi =-\frac{n x^{\sigma }}{\sigma }+\frac{t^{\delta }}{\delta },~\phi =\frac{\alpha x^{\sigma }}{\sigma }-\frac{\beta t^{\delta }}{\delta }. \end{aligned}$$

Here $n,~ \alpha ~{and}~ \beta$ are non-zero free unknowns to be evaluated. By switching (4) into (3), we get

5 $$\begin{aligned} J(V,V',V'',V''',\cdots ) = 0. \end{aligned}$$

### The improved *F*-expansion scheme

The circumstances for the improved *F*-expansion scheme are described in the following phases.

*Ist Step:* The solution of Eq. (1) is presumable in the form that follows the improved *F*-expansion method.

6 $$\begin{aligned} V(\chi ) =\sum _{i=0}^{N}p_i(p +F(\chi ))^i +\sum _{i=1}^{N}q_i(p+F(\chi ))^{-i}, \end{aligned}$$

where either $p_i$ or $q_i$ may be zero, but neither may be zero simultaneously. $q_i (i=1,2,3,\ldots ,N)$ and *p* are fictitious factors that will eventually be chosen, along with $p_i(i=0,1,2,\ldots ,N)$. We consider about popular Riccati equation.

7 $$\begin{aligned} F'(\chi )= m+ F^2(\chi ), \end{aligned}$$

where *m* represents the real part of the equation and the prime represents derivatives with respect to $\chi$. The three general solutions of the Riccati equation Eq. (7) are follows as

*Case-I:* If $m <0$, then the general solutions are

8 $$\begin{aligned} F_1= & {} -\sqrt{-m} \tanh (\sqrt{-m} \chi ), \end{aligned}$$

9 $$\begin{aligned} F_2= & {} -\sqrt{-m} \coth (\sqrt{-m} \chi ). \end{aligned}$$

*Case-II:* If $m >0$, then the general solutions are

10 $$\begin{aligned} F_3= & {} \sqrt{m} \tan (\sqrt{m} \chi ), \end{aligned}$$

11 $$\begin{aligned} F_4= & {} -\sqrt{m} \cot (\sqrt{m} \chi ). \end{aligned}$$

*Case-III:* If $m=0$, then the general solution is

12 $$\begin{aligned} F_5 = -\frac{1}{\chi }. \end{aligned}$$

*2nd step:* The balancing principal is used to gain the value of *N* come out the solution of Eq. ( 5).

*3rd step:* With the help of Eq. (6) togather with Eq. (7) in Eq. (5), it is possible to calculate the polynomial in $F(\chi )$. An algebraic system of equations is therefore produced when the same index of $F(\chi )$ is equal to zero. By using Mathematica to solve these equations, we can get the values of the unknowns $p _i, q_ i$, m, and c, which will be utilized to obtain the answer to Eq. (3).

### The extended simple equation method

We suppose the trial solution of the partial differential equation (PDE) of Eq. (1) that can be expanded in series as follows in order to achieve various results.

13 $$\begin{aligned} v(\chi )=\sum _{i=-N}^{N}b_iR^i(\chi ), \end{aligned}$$

where $b_i\ne 0$ is a constant to be determined later and *N* is a positive integer that can be calculated by applying the balancing principle to Eq. (5). The ordinary differential equation (ODE) is satisfied by $R(\chi )$:

14 $$\begin{aligned} R'(\chi )=c_0 + c_1 R(\chi )+c_2 R^2(\chi ), \end{aligned}$$

where $c_0,c_1,c_2$ are constants. By taking $c_1=0$, Eq. (14) turns to Riccati equation and has solution

15 $$\begin{aligned} R(\chi )= & {} \frac{\sqrt{c_0c_2}}{c_2}tanh(\sqrt{c_0c_2}\chi -\chi _0),~c_0c_2>0. \end{aligned}$$

16 $$\begin{aligned} R(\chi )= & {} -\frac{\sqrt{-c_0c_2}}{c_2}tan(-\sqrt{c_0c_2}(\chi -\frac{s~ln(\chi _0)}{2}),~\chi _0>0,~c_0c_2<0,~s=\pm 1. \end{aligned}$$

by taking $c_0=0$, Eq. (14) turns to Bernoulli equation and has solution

17 $$\begin{aligned} R(\chi )= & {} \frac{c_1 e^{c_1(\chi +\chi _0)}}{1-e^{c_1(\chi +\chi _0)}},~c_1>1, \end{aligned}$$

18 $$\begin{aligned} R(\chi )= & {} -\frac{c_1 e^{c_1(\chi +\chi _0)}}{1+e^{c_1(\chi +\chi _0)}},~c_1<1. \end{aligned}$$

By taking $c_0=0,c_1=0$, Eq. (14) turns to separable equation and has solution

19 $$\begin{aligned} R(\chi )=\frac{1}{-c_2\chi +\chi _0},~c_2\ne 0. \end{aligned}$$

The general solution of Eq. (14) is

20 $$\begin{aligned} R(\chi )= & {} -\frac{c_1-\sqrt{4c_0c_2-c_1^2}tan(\frac{\sqrt{4c_0c_2-c_1^2}}{2}(\chi +\chi _0))}{2c_2},~4c_0c_2>c_1^2,~c_2>0. \end{aligned}$$

21 $$\begin{aligned} R(\chi )= & {} \frac{c_1+\sqrt{4c_0c_2-c_1^2}tan(\frac{\sqrt{4c_0c_2-c_1^2}}{2}(\chi +\chi _0))}{2c_2},~4c_0c_2>c_1^2,~c_2<0, \end{aligned}$$

where $\chi _0$ represent the constant of integration.

We equal to zero all the accumulated factors of powers of $R(\chi )$ after switching Eqs. (13) and (14) into Eq. (5) and performing various calculations. We obtain an algebraic system of equations. It satisfies the requirement that the final solutions of Eq. (3) be determined.

## Extraction of wave solutions

In this section we tackle new traveling-wave solutions for the extended NLSE equation which profoundly relates to superconductivity, plasma physics and non-linear optics. From Eq. (1) with the assistance of Eq. (4) is separated into the following imaginary and real parts:

22 $$\begin{aligned}{} & {} -\gamma _3 V^{'''}+ 6(3 \psi _1+2 \psi _2) V^2 V^{'} + 3(\beta ^2 \gamma _3 +2 \beta \gamma _2 -2 n +2 \gamma _1) V^{'}=0, \end{aligned}$$

23 $$\begin{aligned}{} & {} 6 \left( \beta \psi _1+b\right) V{}^3+ (\beta ^3 \gamma _3 +3 \beta ^2 \gamma _2 +6 (\beta \gamma _1 -\alpha )) V-3 \left( \beta \gamma _3+\gamma _2\right) V''=0. \end{aligned}$$

The Eq. (23) is the nonlinear ordinary differential equation of Eq. (1), and Eq. (22) is integrated with $\chi$, with zero as the integration constant. So, the Eq. (22) becomes,

24 $$\begin{aligned} 2(3 \psi _1 +2 \psi _2 )V^3+3( \beta ^2 \gamma _3 +2( \beta \gamma _2 - n + \gamma _1)) V-\gamma _3 V''=0. \end{aligned}$$

The following proportion is written using the homogeneous balance between Eqs. (23) and (24):

25 $$\begin{aligned} \frac{3(\beta \psi _1+b)}{3\psi _1+2\psi ^2}=\frac{\beta ^3\gamma _3+3\beta ^2 \gamma _2+ \beta \gamma _1-6\alpha }{3(\beta ^2\gamma _3+2(\beta \gamma _2-n+\gamma _1))}=\frac{3(\beta \gamma _3+\gamma _2)}{\gamma _3}. \end{aligned}$$

From the aforementioned proposition, one can arrive at the following constraints:

26 $$\begin{aligned} n= & {} \frac{4 \beta ^3 \gamma ^2_3+ 12 \beta ^2 \gamma _2 \gamma _3 +6 \beta \gamma _1 \gamma _3 +9 \beta \gamma ^2_2 + 3 \alpha \gamma _3 +9 \gamma _1 \gamma _2}{9(\beta \gamma _3 +\gamma _2)}, ~ \gamma _2,~\gamma _3 \ne 0. \end{aligned}$$

27 $$\begin{aligned} b= & {} \frac{2 \gamma _2(\beta \psi _1 +\beta \psi _2)+\gamma _2(3\psi _1 +2 \psi _2)}{\gamma _3},~ ~ \gamma _3 \ne 0. \end{aligned}$$

### Application of improved *F*-expansion method

Following the balancing principle of the terms $V^{''}$ and $V^3$ in Eq. (23), we obtain *N* = 1. Now, by combining Eq. (6) and the solutions of Eq. (7) in Eq. (23) and then using Mathematica, we obtain

28 $$\begin{aligned} 6 b q_1^3-6 \beta \gamma _3 m^2 q_1-6 \gamma _2 m^2 q_1+6 \beta q_1^3 \psi _1= & {} 0,\nonumber \\ 18 b p_0 p_1^2+18 \beta p_0 p_1^2 \psi _1= & {} 0,\nonumber \\ 6 b p_1^3-6 \beta \gamma _3 p_1+6 \beta p_1^3 \psi _1-6 \gamma _2 p_1= & {} 0,\nonumber \\ 36 b p_0 p_1 q_1+6 b p_0^3-6 \alpha p_0+\beta ^3 \gamma _3 p_0+3 \beta ^2 \gamma _2 p_0+6 \beta \gamma _1 p_0+6 \beta p_0^3 \psi _1+36 \beta p_0 p_1 q_1 \psi _1= & {} 0,\nonumber \\ 18 b p_1^2 q_1+18 b p_0^2 p_1-6 \beta \gamma _3 m p_1-6 \gamma _2 m p_1-6 \alpha p_1+\beta ^3 \gamma _3 p_1+3 \beta ^2 \gamma _2 p_1+6 \beta \gamma _1 p_1+18 \beta p_0^2 p_1 \psi _1+18 \beta p_1^2 q_1 \psi _1= & {} 0,\nonumber \\ 18 b p_0 q_1^2+18 \beta p_0 q_1^2 \psi _1= & {} 0,\nonumber \\ 18 b p_1 q_1^2+18 b p_0^2 q_1-6 \beta \gamma _3 m q_1-6 \gamma _2 m q_1+18 \beta p_1 q_1^2 \psi _1+18 \beta p_0^2 q_1 \psi _1-6 \alpha q_1+\beta ^3 \gamma _3 q_1+3 \beta ^2 \gamma _2 q_1+6 \beta \gamma _1 q_1= & {} 0. \end{aligned}$$

*Case-I:*

29 $$\begin{aligned} p_0=0,~p_1=-\frac{\sqrt{\beta \gamma _3+\gamma _2}}{\sqrt{\beta \psi _1+b}},~q_1=-\frac{m \sqrt{\beta \gamma _3+\gamma _2}}{\sqrt{\beta \psi _1+b}},~\alpha =\frac{1}{6} \left( \beta ^3 \gamma _3+3 \beta ^2 \gamma _2+6 \beta \gamma _1+12 \beta \gamma _3 m+12 \gamma _2 m\right) . \end{aligned}$$

when $m>0$, the solutions are

*Family 1*

30 $$\begin{aligned} v_1(x,t)=-\frac{2 \sqrt{m} e^{i \phi } \sqrt{\beta \gamma _3+\gamma _2} \csc \left( 2 \sqrt{m} \chi \right) }{\sqrt{\beta \psi _1+b}}. \end{aligned}$$

*Family 2*

31 $$\begin{aligned} v_2(x,t)=\frac{2 \sqrt{m} e^{i \phi } \sqrt{\beta \gamma _3+\gamma _2} \csc \left( 2 \sqrt{m} \chi \right) }{\sqrt{\beta \psi _1+b}}. \end{aligned}$$

When $m<0$, the solutions are

*Family 3*

32 $$\begin{aligned} v_3(x,t)=-\frac{2 \sqrt{-m} e^{i \phi } \sqrt{\beta \gamma _3+\gamma _2} \text {csch}\left( 2 \sqrt{-m} \chi \right) }{\sqrt{\beta \psi _1+b}}. \end{aligned}$$

*Family 4*

33 $$\begin{aligned} v_4(x,t)=\frac{\sqrt{-m} e^{i \phi } \sqrt{\beta \gamma _3+\gamma _2} \left( \text {coth}\left( \sqrt{-m} \chi \right) ^2-1\right) }{\sqrt{\beta \psi _1+b} \text {coth}\left( \sqrt{-m} \chi \right) }. \end{aligned}$$

When $m=0$, the solutions are

*Family 5*

34 $$\begin{aligned} v_5(x,t)=\frac{e^{i \phi } \sqrt{\beta \gamma _3+\gamma _2} \left( m \chi ^2+1\right) }{\chi \sqrt{\beta \psi _1+b}}. \end{aligned}$$

For case-I $$\chi =\frac{t^{\delta }}{\delta }-\frac{n x^{\sigma }}{\sigma }, \psi = \frac{x^{\sigma } \left( \beta ^3 \gamma _3+3 \beta ^2 \gamma _2+6 \beta \gamma _1+12 \beta \gamma _3 m+12 \gamma _2 m\right) }{6 \sigma }-\frac{\beta t^{\delta }}{\delta }$$.

*Case-II:*

35 $$\begin{aligned} p_0=0,~p_1=\frac{\sqrt{2} \sqrt{-3 \alpha +\beta ^2 \gamma _2+3 \beta \gamma _1}}{\sqrt{\beta ^3 \left( -\psi _1\right) -b \beta ^2+6 b m+6 \beta m \psi _1}},~q_1=0,~\gamma _3=\frac{3 \left( -2 \alpha +\beta ^2 \gamma _2+2 \beta \gamma _1-2 \gamma _2 m\right) }{6 \beta m-\beta ^3}. \end{aligned}$$

When $m>0$, the solutions are

*Family 1*

36 $$\begin{aligned} v_6(x,t)=\frac{\sqrt{2} \sqrt{m} e^{i \phi } \sqrt{-3 \alpha +\beta ^2 \gamma _2+3 \beta \gamma _1} \tan \left( \sqrt{m} \chi \right) }{\sqrt{\left( 6 m-\beta ^2\right) \left( \beta \psi _1+b\right) }}. \end{aligned}$$

*Family 2*

37 $$\begin{aligned} v_7(x,t)=-\frac{\sqrt{2} \sqrt{m} e^{i \phi } \sqrt{-3 \alpha +\beta ^2 \gamma _2+3 \beta \gamma _1} \cot \left( \sqrt{m} \chi \right) }{\sqrt{\left( 6 m-\beta ^2\right) \left( \beta \psi _1+b\right) }}. \end{aligned}$$

When $m<0$, the solutions are

*Family 3*

38 $$\begin{aligned} v_8(x,t)=-\frac{\sqrt{2} \sqrt{-m} e^{i \phi } \sqrt{-3 \alpha +\beta ^2 \gamma _2+3 \beta \gamma _1} \tanh \left( \sqrt{-m} \chi \right) }{\sqrt{\left( 6 m-\beta ^2\right) \left( \beta \psi _1+b\right) }}. \end{aligned}$$

*Family 4*

39 $$\begin{aligned} v_9(x,t)=-\frac{\sqrt{2} \sqrt{-m} e^{i \phi } \sqrt{-3 \alpha +\beta ^2 \gamma _2+3 \beta \gamma _1} \coth \left( \sqrt{-m} \chi \right) }{\sqrt{\left( 6 m-\beta ^2\right) \left( \beta \psi _1+b\right) }}. \end{aligned}$$

When $m=0$, the solutions are

*Family 5*

40 $$\begin{aligned} v_{10}(x,t)=-\frac{e^{i \phi } \sqrt{-6 \alpha +2 \beta ^2 \gamma _2+6 \beta \gamma _1}}{\chi \sqrt{\left( 6 m-\beta ^2\right) \left( \beta \psi _1+b\right) }}. \end{aligned}$$

For case-II $\chi =\frac{t^{\delta }}{\delta }-\frac{n x^{\sigma }}{\sigma } ~ and~\phi =\frac{\alpha x^{\sigma }}{\sigma }-\frac{\beta t^{\delta }}{\delta }.$

### Application of extended simple equation method

Switching the Eq. (13) togather with the solutions of Eq. (14) in Eq. (23) and then using Mathematica, we get

41 $$\begin{aligned} 6 b_{-1}^3 \left( b+\psi _1\right) -6 b_{-1} c_0^2 \left( \gamma _2+\gamma _3\right)= & {} 0,\nonumber \\ 9 b_{-1} \left( 2 b_{-1} b_0 \left( b+\psi _1\right) -c_0 c_1 \left( \gamma _2+\gamma _3\right) \right)= & {} 0,\nonumber \\ b_{-1} \left( 18 b_0^2 \left( b+\psi _1\right) +18 b_{-1} b_1 \left( b+\psi _1\right) -3 c_1^2 \gamma _2-6 c_0 c_2 \gamma _2-3 c_1^2 \gamma _3-6 c_0 c_2 \gamma _3+6 \gamma _1+3 \gamma _2+\gamma _3-6\right)= & {} 0,\nonumber \\ b_1 \left( 18 b_0^2 \left( b+\psi _1\right) +18 b_{-1} b_1 \left( b+\psi _1\right) -3 c_1^2 \gamma _2-6 c_0 c_2 \gamma _2-3 c_1^2 \gamma _3-6 c_0 c_2 \gamma _3+6 \gamma _1+3 \gamma _2+\gamma _3-6\right)= & {} 0,\nonumber \\ 9 b_1 \left( 2 b_0 b_1 \left( b+\psi _1\right) -c_1 c_2 \left( \gamma _2+\gamma _3\right) \right)= & {} 0,\nonumber \\ 6 \left( b_1^3 \left( b+\psi _1\right) -b_1 c_2^2 \left( \gamma _2+\gamma _3\right) \right)= & {} 0,\nonumber \\ -3 c_1 \left( \gamma _2+\gamma _3\right) \left( b_1 c_0+b_{-1} c_2\right) +b_0 \left( 36 b_{-1} b_1 \left( b+\psi _1\right) +6 \gamma _1+3 \gamma _2+\gamma _3-6\right) +6 b_0^3 \left( b+\psi _1\right)= & {} 0. \end{aligned}$$

*Case-I:*

42 $$\begin{aligned} b_{-1}= & {} 0,~b_1=-\frac{i c_2 \sqrt{\gamma _2+\gamma _3}}{\sqrt{-b-\psi _1}},~~c_0=\frac{6 b_0^2 \psi _1+6 b b_0^2+6 \gamma _1+3 \gamma _2+\gamma _3-6}{6 c_2 \left( \gamma _2+\gamma _3\right) },\nonumber \\ c_1= & {} \frac{2 \left( -\frac{i b b_0 \sqrt{\gamma _2+\gamma _3}}{\sqrt{-b-\psi _1}}-\frac{i b_0 \sqrt{\gamma _2+\gamma _3} \psi _1}{\sqrt{-b-\psi _1}}\right) }{\gamma _2+\gamma _3},~\nonumber \\ \gamma _1= & {} \frac{1}{6} \left( -6 b_0^2 \psi _1-6 b b_0^2+6 c_0 c_2 \gamma _2+6 c_0 c_2 \gamma _3-3 \gamma _2-\gamma _3+6\right) . \end{aligned}$$

If we take $c_1=0$, using Eqs. (40) and (13), we finally arrive at the solutions for Eq. (1) given by

*Family 1*

43 $$\begin{aligned} v_1(x,t)=e^{i \phi } \left( b_0-\frac{i \sqrt{c_0 c_2} \sqrt{\gamma _2+\gamma _3} \tan (\sqrt{c_0 c_2} \left( \chi )\right) }{\sqrt{-b-\psi _1}}\right) . \end{aligned}$$

*Family 2*

44 $$\begin{aligned} v_2(x,t)=e^{i \phi } \left( b_0-\frac{i \sqrt{-c_0 c_2} \sqrt{\gamma _2+\gamma _3} \tanh \left( \frac{1}{2} s ln \left( \chi _0\right) -\sqrt{-c_0 c_2} \chi \right) }{\sqrt{-b-\psi _1}}\right) . \end{aligned}$$

When $c_0=0$, the solutions are

*Family 3*

45 $$\begin{aligned} v_3(x,t)=e^{i \phi } \left( b_0-\frac{2 i c_2 \left( -\frac{i b b_0 \sqrt{\gamma _2+\gamma _3}}{\sqrt{-b-\psi _1}}-\frac{i b_0 \sqrt{\gamma _2+\gamma _3} \psi _1}{\sqrt{-b-\psi _1}}\right) \exp \left( \frac{2 \left( -\frac{i b b_0 \sqrt{\gamma _2+\gamma _3}}{\sqrt{-b-\psi _1}}-\frac{i b_0 \sqrt{\gamma _2+\gamma _3} \psi _1}{\sqrt{-b-\psi _1}}\right) \left( i\chi \right) }{\gamma _2+\gamma _3}\right) }{\sqrt{\gamma _2+\gamma _3} \sqrt{-b-\psi _1} \left( 1-c_2 \exp \left( \frac{2 \left( -\frac{i b b_0 \sqrt{\gamma _2+\gamma _3}}{\sqrt{-b-\psi _1}}-\frac{i b_0 \sqrt{\gamma _2+\gamma _3} \psi _1}{\sqrt{-b-\psi _1}}\right) \left( \chi \right) }{\gamma _2+\gamma _3}\right) \right) }\right) . \end{aligned}$$

*Family 4*

46 $$\begin{aligned} v_4(x,t)=e^{i \phi } \left( b_0+\frac{2 i c_2 \left( -\frac{i b b_0 \sqrt{\gamma _2+\gamma _3}}{\sqrt{-b-\psi _1}}-\frac{i b_0 \sqrt{\gamma _2+\gamma _3} \psi _1}{\sqrt{-b-\psi _1}}\right) \exp \left( \frac{2 \left( -\frac{i b b_0 \sqrt{\gamma _2+\gamma _3}}{\sqrt{-b-\psi _1}}-\frac{i b_0 \sqrt{\gamma _2+\gamma _3} \psi _1}{\sqrt{-b-\psi _1}}\right) \left( \chi \right) }{\gamma _2+\gamma _3}\right) }{\sqrt{\gamma _2+\gamma _3} \sqrt{-b-\psi _1} \left( 1+c_2 \exp \left( \frac{2 \left( -\frac{i b b_0 \sqrt{\gamma _2+\gamma _3}}{\sqrt{-b-\psi _1}}-\frac{i b_0 \sqrt{\gamma _2+\gamma _3} \psi _1}{\sqrt{-b-\psi _1}}\right) \left( \chi \right) }{\gamma _2+\gamma _3}\right) \right) }\right) . \end{aligned}$$

When $c_0,c_1,c_2$, the solutions of Eq. (1) are

*Family 5*

47 $$\begin{aligned} v_5(x,t)=e^{i \phi } \left( b_0+\frac{i \sqrt{\gamma _2+\gamma _3} \left( c_1-\sqrt{4 c_0 c_2-c_1^2} \tan \left( \frac{1}{2} \sqrt{4 c_0 c_2-c_1^2} \left( \chi +\chi _0\right) \right) \right) }{2 \sqrt{-b-\psi _1}}\right) . \end{aligned}$$

*Family 6*

48 $$\begin{aligned} v_6(x,t)=e^{i \phi } \left( b_0-\frac{i \sqrt{\gamma _2+\gamma _3} \left( \sqrt{4 c_0 c_2-c_1^2} \tan \left( \frac{1}{2} \sqrt{4 c_0 c_2-c_1^2} \left( \chi +\chi _0\right) \right) +c_1\right) }{2 \sqrt{-b-\psi _1}}\right) . \end{aligned}$$

*Case-II:*

49 $$\begin{aligned} b_0=\frac{\sqrt{6 c_0 c_2 \gamma _2+6 c_0 c_2 \gamma _3-6 \gamma _1-3 \gamma _2-\gamma _3+6}}{\sqrt{6} \sqrt{b+\psi _1}},~~b_{-1}=0,~~b_1=\frac{i c_2 \sqrt{\gamma _2+\gamma _3}}{\sqrt{-b-\psi _1}},~~~~~~~~~~~~~~~~~~~~\nonumber \\c_1=\frac{\frac{i \sqrt{\frac{2}{3}} \sqrt{\gamma _2+\gamma _3} \psi _1 \sqrt{6 c_0 c_2 \gamma _2+6 c_0 c_2 \gamma _3-6 \gamma _1-3 \gamma _2-\gamma _3+6}}{\sqrt{-b-\psi _1} \sqrt{b+\psi _1}}+\frac{i \sqrt{\frac{2}{3}} b \sqrt{\gamma _2+\gamma _3} \sqrt{6 c_0 c_2 \gamma _2+6 c_0 c_2 \gamma _3-6 \gamma _1-3 \gamma _2-\gamma _3+6}}{\sqrt{-b-\psi _1} \sqrt{b+\psi _1}}}{\gamma _2+\gamma _3}. \end{aligned}$$

If we take $c_1=0$, using Eqs. (40) and (13), we finally arrive at the solutions for Eq. (1) given by

*Family 1*

50 $$\begin{aligned} v_{7}(x,t)=e^{i \phi } \left( \frac{\sqrt{6 c_0 c_2 \gamma _2+6 c_0 c_2 \gamma _3-6 \gamma _1-3 \gamma _2-\gamma _3+6}}{\sqrt{6} \sqrt{b+\psi _1}}+\frac{i \sqrt{c_0 c_2} \sqrt{\gamma _2+\gamma _3} \tan \left( \sqrt{c_0 c_2} \left( \chi \right) \right) }{\sqrt{-b-\psi _1}}\right) . \end{aligned}$$

*Family 2*

51 $$\begin{aligned} v_{8}(x,t)=e^{i \phi } \left( \frac{\sqrt{6 c_0 c_2 \gamma _2+6 c_0 c_2 \gamma _3-6 \gamma _1-3 \gamma _2-\gamma _3+6}}{\sqrt{6} \sqrt{b+\psi _1}}+ \frac{i \sqrt{-c_0 c_2} \sqrt{\gamma _2+\gamma _3} \tanh \left( \frac{1}{2} s \log \left( \chi \right) -\sqrt{-c_0 c_2} \left( \chi +\chi _0 \right) \right) }{\sqrt{-b-\psi _1}}\right) . \end{aligned}$$

When $c_0=0$, the solutions are

*Family 3*

52 $$\begin{aligned} v_{9}(x,t)=e^{i \phi } \left( b_0+\frac{i \sqrt{\gamma _2+\gamma _3} c_1 c_2 e^{c_1 \chi }}{\sqrt{-b-\psi _1} \left( 1-c_2 e^{c_1 \chi }\right) }\right) . \end{aligned}$$

*Family 4*

53 $$\begin{aligned} v_{10}(x,t)=e^{i \phi } \left( b_0-\frac{i c_1 c_2 \sqrt{\gamma _2+\gamma _3} e^{c_1 \chi }}{\sqrt{-b-\psi _1} \left( c_2 e^{c_1 \chi }+1\right) }\right) . \end{aligned}$$

When $c_0,c_1,c_2$, the solutions of Eq. (1) are

*Family 5*

54 $$\begin{aligned} v_{11}(x,t)=e^{i \phi } \left( b_0-\frac{i \sqrt{\gamma _2+\gamma _3} \left( c_1-\sqrt{4 c_0 c_2-c_1^2} \tan \left( \frac{1}{2} \sqrt{4 c_0 c_2-c_1^2} \left( \chi +\chi _0\right) \right) \right) }{2 \sqrt{-b-\psi _1}}\right) . \end{aligned}$$

*Family 6*

55 $$\begin{aligned} v_{12}(x,t)=e^{i \phi } \left( b_0+\frac{i \sqrt{\gamma _2+\gamma _3} \left( \sqrt{4 c_0 c_2-c_1^2} \tan \left( \frac{1}{2} \sqrt{4 c_0 c_2-c_1^2} \left( \chi +\chi _0\right) \right) +c_1\right) }{2 \sqrt{-b-\psi _1}}\right) . \end{aligned}$$

For case-I and case-II $\chi =-\frac{n x^{\sigma }}{\sigma }+\frac{t^{\delta }}{\delta }~and~\phi =\frac{\beta t^{\delta }}{\delta }+\frac{\alpha x^{\sigma }}{\sigma }.$

## Stability analysis

This section examines the stability of the computed solutions to demonstrate their suitability for model applications. Using the characteristics of the Hamiltonian system to handle Eq. (36) under the following conditions $[m=2,~\alpha =3,~\gamma _1=3,~\gamma _2=1,~b=4,~\beta =1.3,~\psi _1=0.2]$ in momentum having the following form:

56 $$\begin{aligned} N=\frac{1}{2}\int _{-\infty }^{\infty }v^2~dn. \end{aligned}$$

Thus, *N* represents momentum and *v* represents the potential of the electric field. The prerequisite for soliton stability is

57 $$\begin{aligned} \frac{\partial {N}}{\partial {n}}~>~0, \end{aligned}$$

where *n* is the wave velocity. As a result, the following formulation is used to investigate the stability property of Eq. (36).

58 $$\begin{aligned} N=\frac{0.141355 \left( -\tan ^{-1}\left( \tan \left( \sqrt{2} (10 n+1)\right) \right) +\tan ^{-1}\left( \tan \left( \sqrt{2} (1-10 n)\right) \right) +\tan \left( \sqrt{2} (10 n+1)\right) -\tan \left( \sqrt{2} (1-10 n)\right) \right) }{n}. \end{aligned}$$

Thus, we find

59 $$\begin{aligned} \frac{\partial {N}}{\partial {n}}|_{c=5}=0.0204671. \end{aligned}$$

Thus, Eq. (36) is a stable solution in $x \in ~[-10,10]$. The stability conditions of different solutions can be studied using the same strategy.

## Results and discussions

**Figure 1:**
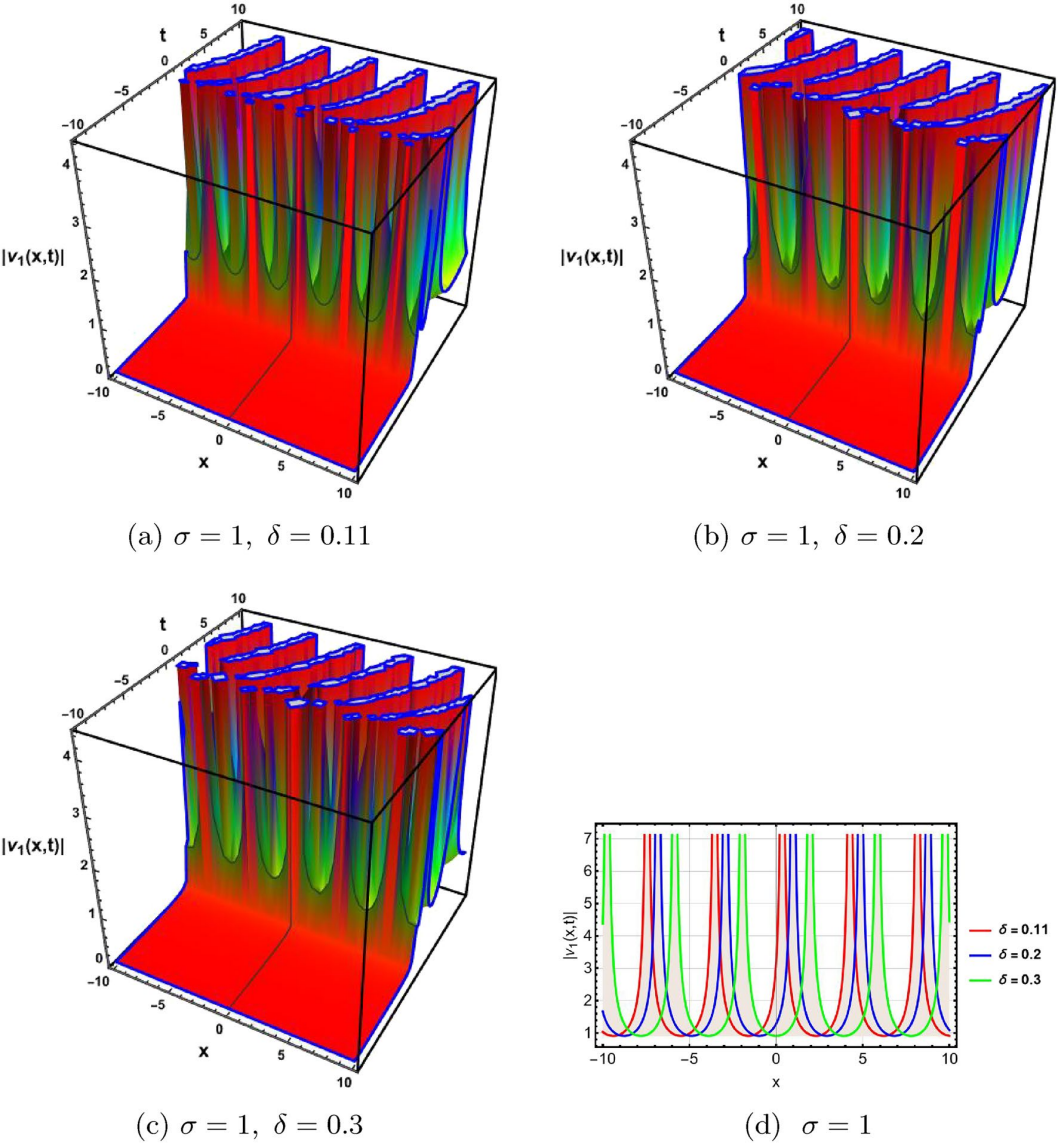
3D, 2D graphs of Eq. (30).

**Figure 2:**
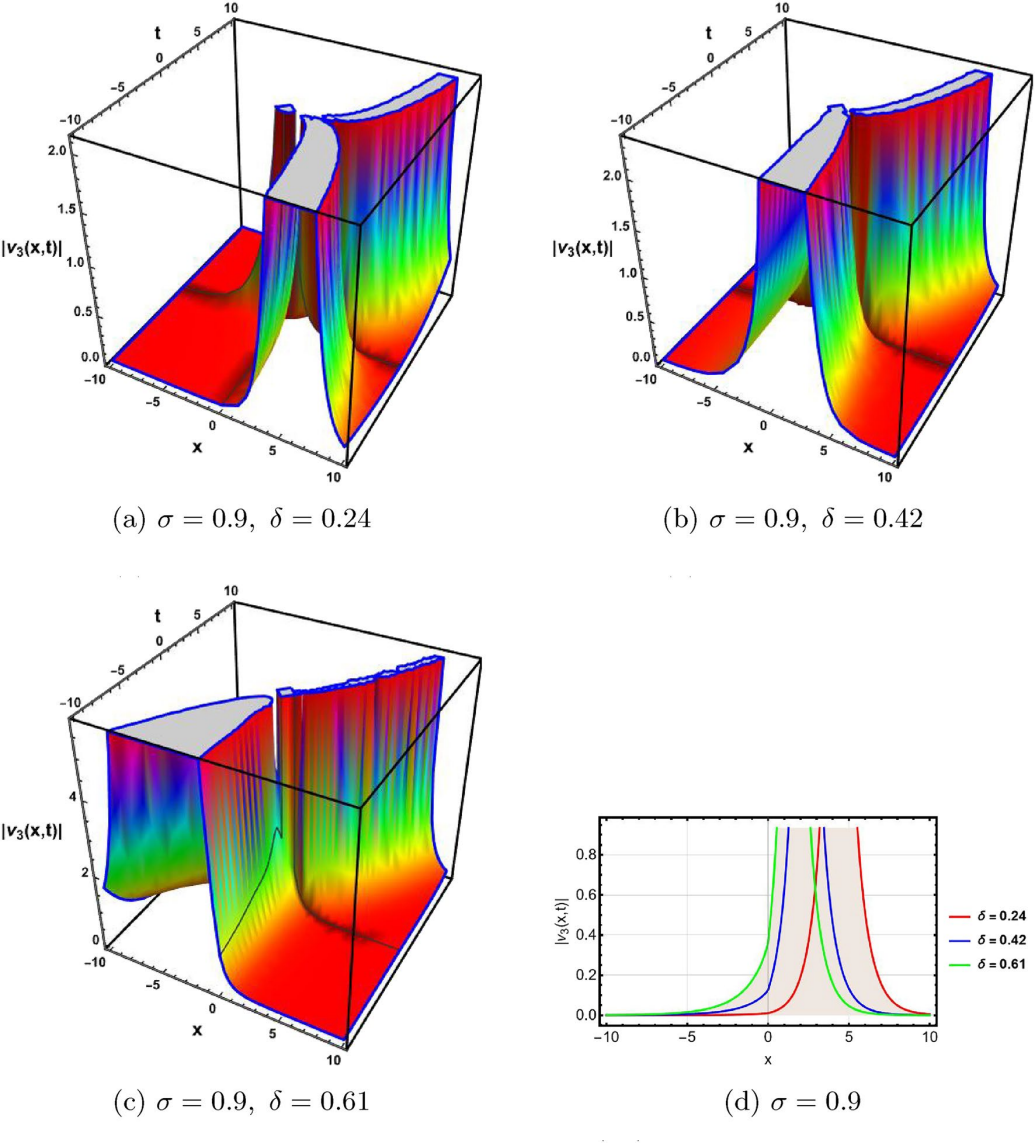
3D, 2D graphs of Eq. (32).

**Figure 3:**
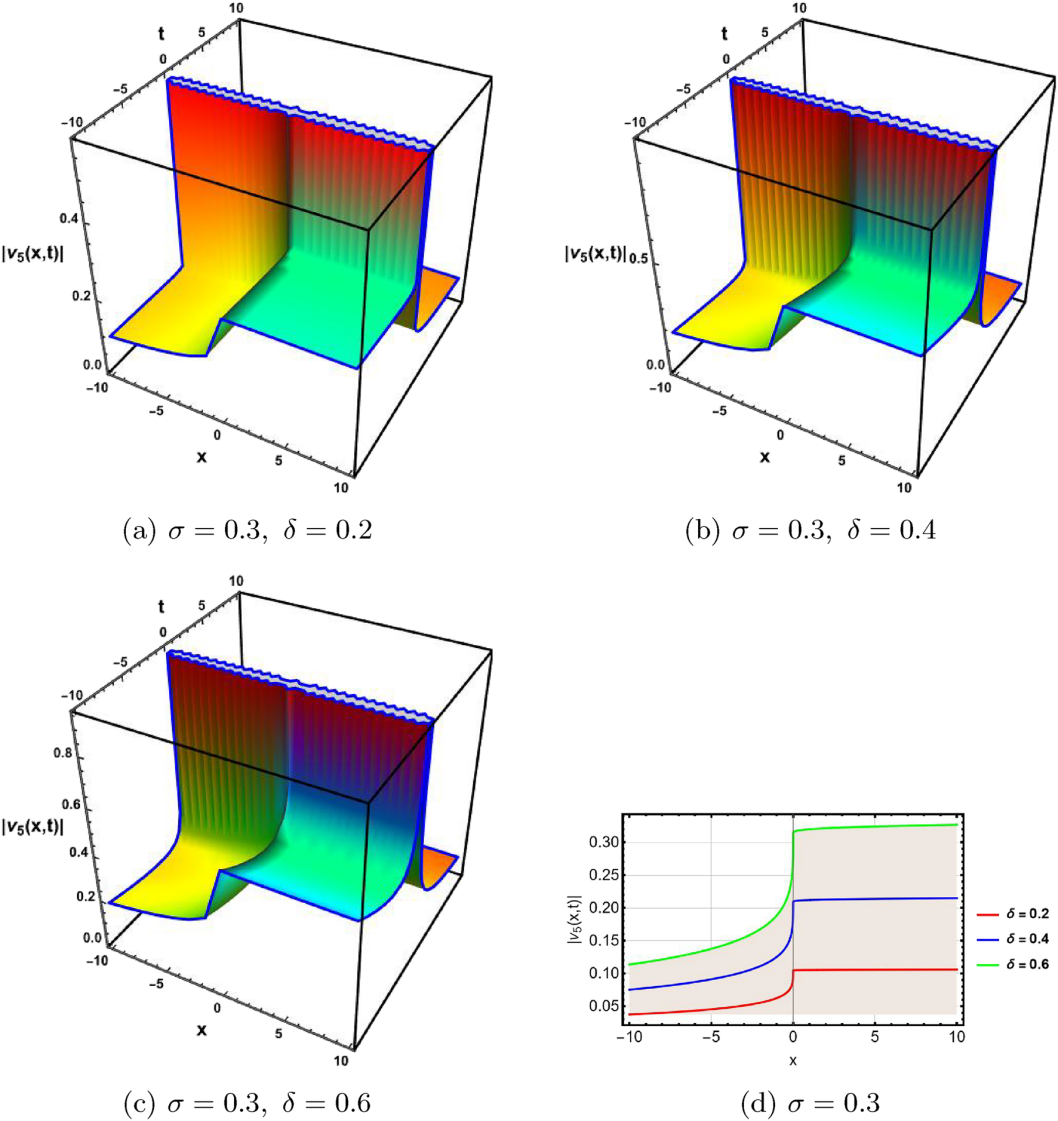
3D, 2D graphs of Eq. (34).

**Figure 4:**
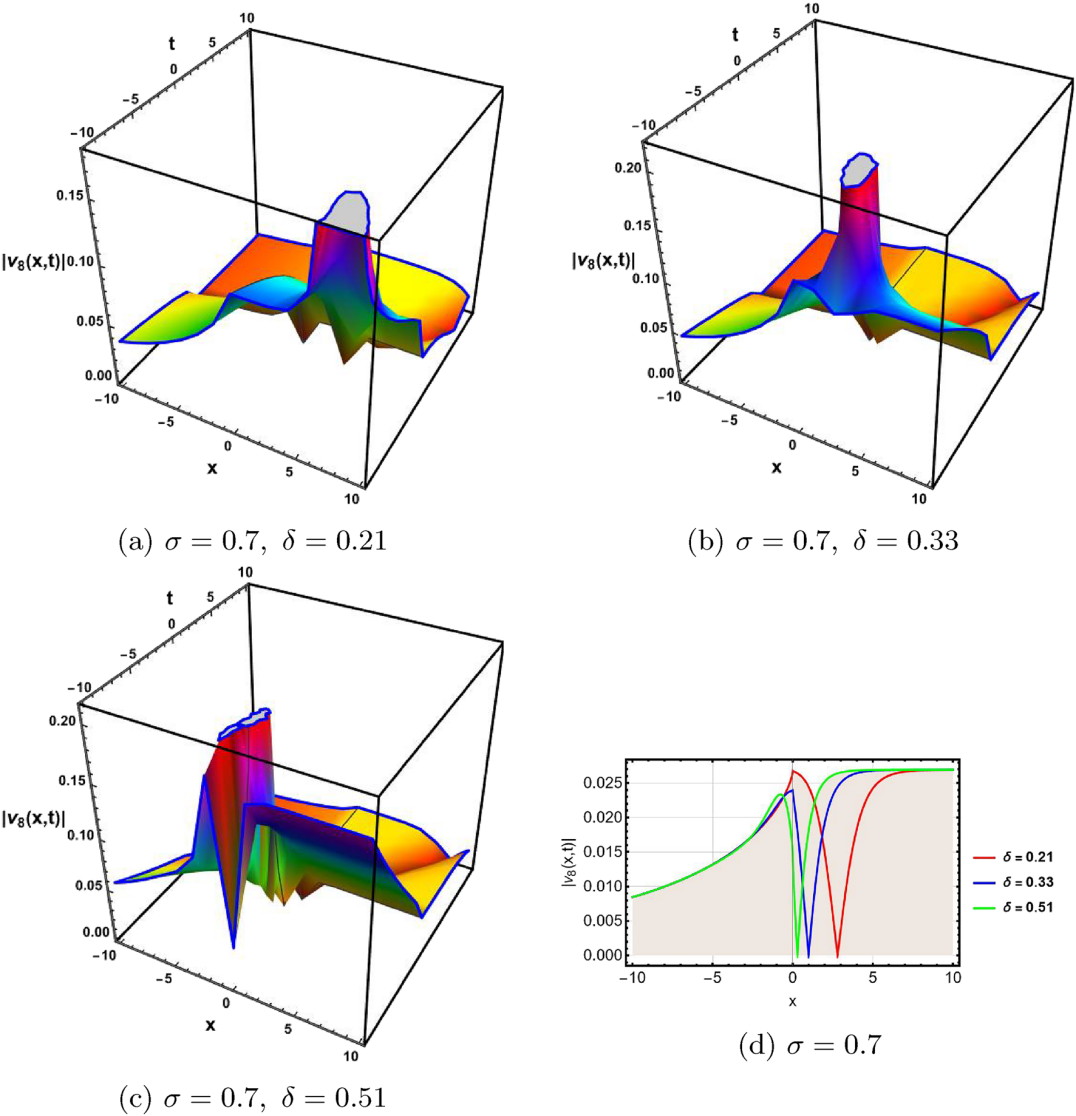
3D, 2D graphs of Eq. (38).

**Figure 5:**
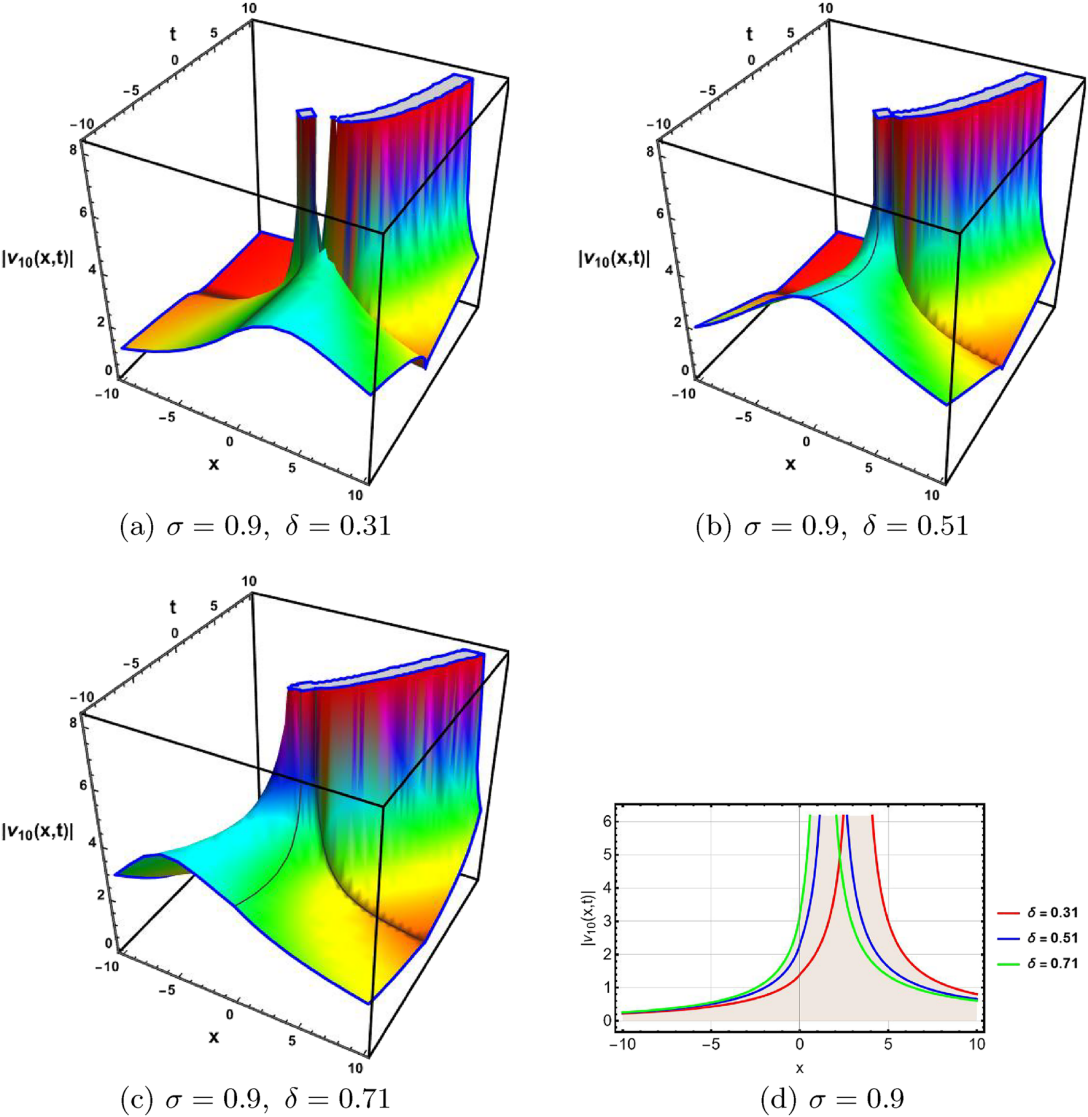
3D, 2D graphs of Eq. (40).

**Figure 6:**
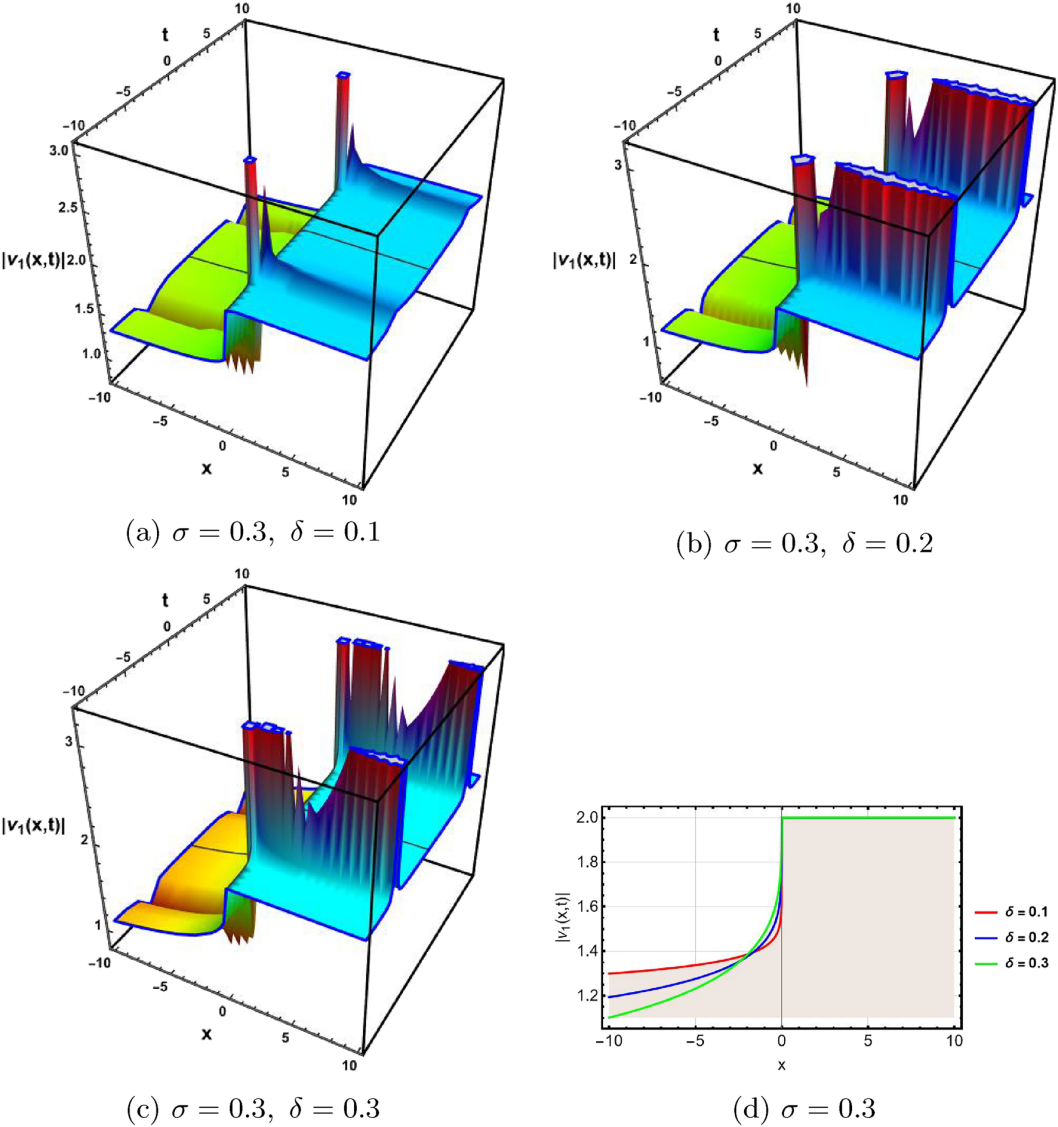
3D, 2D graphs of Eq. (43).

**Figure 7:**
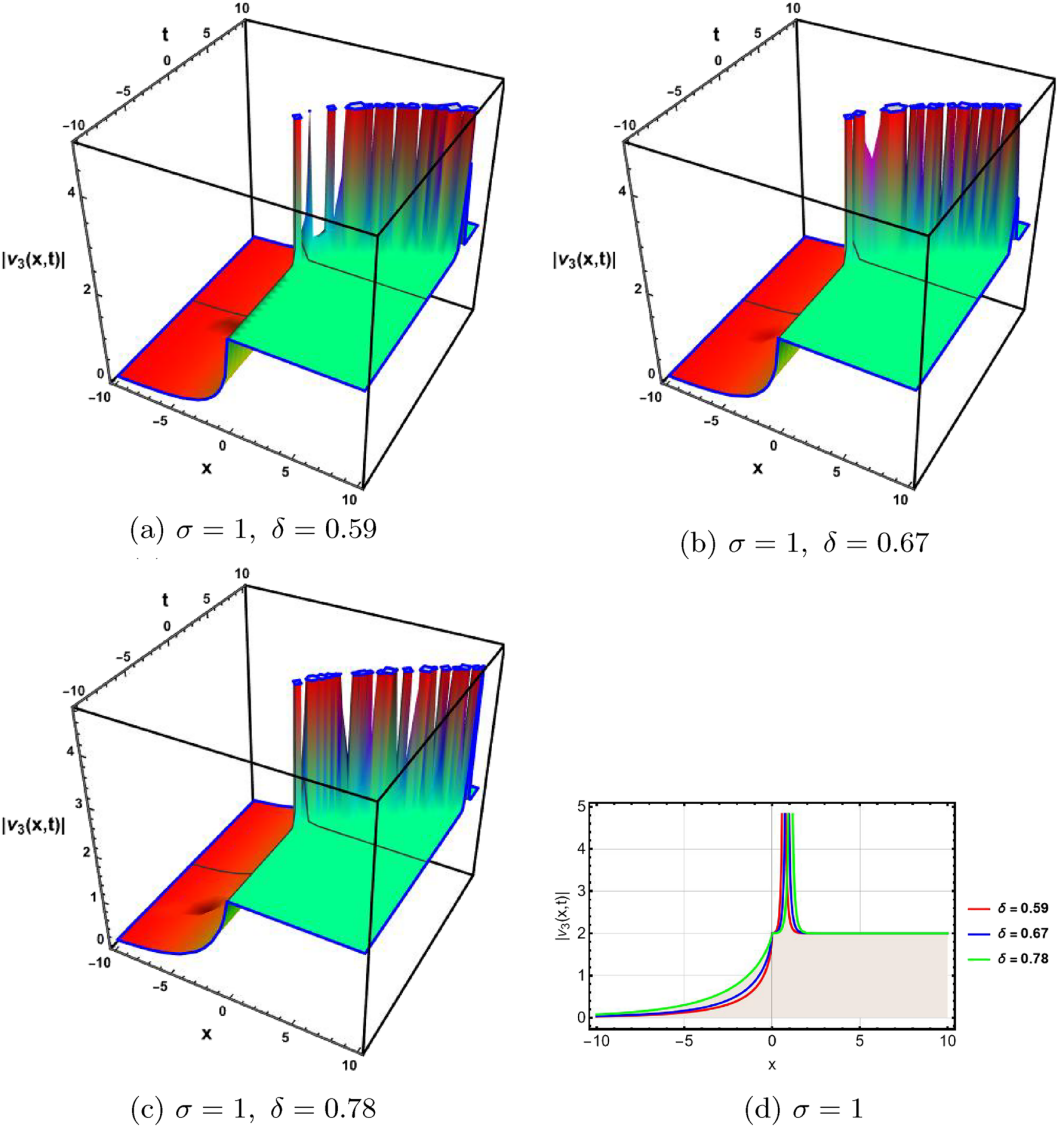
3D, 2D of Eq. (45).

In this section, a comprehensive comparison of the evaluated results is made with the existing computed outcomes, which highlights the novelty of the current study. It is noticed that Ozisik et al.^61^ calculated only a few numbers of solutions by using the modified *F*-expansion method. But we have constructed an abundance of traveling wave solutions in this article by using the extended simple equation approach and the improved *F*-expansion method. Several of our outcomes diverge from those mentioned in^61^ if we compare our achievement with their results. Even so. if we give various values to the components involved, we can obtain some similar outcomes. It is crucial to note that the achievements of this article are practical, compact, eloquent, and straightforward to understand when it comes to nonlinear wave applications. It could also be used in plasma physics, semiconductor materials, optical fiber communications, ultrashort pulses, and other nonlinear optical phenomena, etc. In order to illustrate the relationship between two or more variables in a data set using a graph, a plot is a graphical process. Graphical description is a crucial technique for accurately representing nonlinear events. Plots of the nonlinear equation solutions are crucial for revealing the internal dynamics of various nonlinear processes. The numerical simulations of a few solutions that were discovered are provided in this section by selecting appropriate values for arbitrary parameters. The soliton has the ability to keep its amplitude, velocity, and form constant throughout its propagation. These reported solutions have some physical meaning for instance dark soliton is a soliton whose intensity is lower than the background and which isn’t produced by a typical pulse but rather is basically devoid of energy in a continuous time beam. There are further types of solitary waves called singular solitons that have singularities, typically infinite discontinuities. Singular solitons might be linked to solitary waves when the location of the center of the solitary wave is imaginary. Therefore, discussing the topic of singular solitons is relevant. This type of solution contains spikes and therefore may recommend a description for the development of rogue waves. Periodic wave solution describes a wave with repeating continuous pattern, which determines its wavelength and frequency, while period defines as time required to complete cycle of waveform and frequency is a number of cycles per second of time. Here, we plotted various wave profiles that were extracted from the solutions to Eq. (1) and are shown in 3D and 2D. The Fig. 1 shows the periodic behavior Eq. (30), by choosing the arbitrary parameter values for $~m=0.5,~\psi _1=1.5,~n=0.57,~\gamma _2=0.57,~\gamma _3=0.5,~b=1.3,\beta =0.3,~\gamma _1=0.67$. The Fig. 2 illustrates the singular behavior of the modulus of Eq. (32), by choosing the arbitrary parameter values for $~m=-0.5,~\psi _1=1.4,~n=1,\gamma _2=2,~\gamma _3=3,~b=1.3,~\beta =0.3,~\gamma _1=2$. The Fig. 3 illustrates the rational behavior of Eq. (34), by choosing the arbitrary parameter values for $~m=0,~\psi _1=1.4,~n=0.01,~\gamma _2=0.2,~\gamma _3=0.9,~b=1.3,~\beta =0.3,~\gamma _1=0.6$. The Fig. 4, illustrates the dark behavior of Eq. (38), by choosing the arbitrary parameter values for $~m=-1,~\psi _1=0.2,~n=1,~\gamma _2=0.7,~\gamma _3=0.2,~b=1.3,~\beta =0.3,~\gamma _1=0.6,~\alpha =0.2$. The Fig. 5, illustrates the rational behavior of Eq. (40), by choosing the arbitrary parameter values for $~m=0,~\psi _1=2,~n=1,~\gamma _2=3,~\gamma _3=2,~b=3,~\beta =0.3,~\gamma _1=4,~\alpha =0.2$. The Fig. 6, illustrates the periodic behavior of Eq. (43), by choosing the arbitrary parameter values for $c_1=0,~c_0=0.3,~c_2=0.2,~\gamma _3=0.02,~\gamma _2=-0.04,~\psi _1=0.1,~b=0.12,~n=0.1,~\alpha =0.11,~\beta =-1,~b_0=2,~\chi _0=0$. The Fig. 7, illustrates the exponential behavior of Eq. (45), by choosing the arbitrary parameter values for$c_1=0,~c_0=-0.9,~c_2=0.2,~s=1,~\psi _1=0.4,~b=2,\alpha =0.2,~\beta =0.4,~n=1,~b_0=2,~\gamma _2=1,~\gamma _3=-2,~\chi _0=0.1$. The 2D wave profiles of the obtained solutions have been sketched for various values of $\sigma$ and $\delta$ to demonstrate the effect of the fractional derivative on the dynamic behavior of the waves. From the figures, it is observed that the fractional order has a significant impact on the characteristics of the wave profiles via the memory effect phenomenon, which means that the signal takes into account its past evolution at any point; acting on this parameter allows having better and more complete information about the shape of a signal or a pulse.

## Conclusion

In this paper, we study the improved *F*-expansion and extended simple equation techniques to obtain the soliton solutions of modified extended NLSE with conformable fractional derivatives arising in mono-mode optical fibers. The somatic perspective of the derived wave solutions is illustrated in Figs. 1. 2, 3, 4, 5, 6 and 7 which is useful to comprehend the visuals of solitons and the effects of the third-order dispersion component. Also we obtained solutions of Eq. (1) in the form of exponential, rational, periodic, hyperbolic, and trigonometric functions. The outcomes are a collection of new, extended NLSE solutions, where the suggested methods showed to be more reliable, accurate, and effective. The obtained results and figures Figs. 1, 2, 3, 4, 5, 6 and 7 conclude that, the fractional parameter $\sigma$ and $\delta$ plays the main rule in the solutions. We believe that the travelling wave solutions obtained in this paper should have significant applications in the field of sciences such as plasma physics and compact astronomical phenomena such as femtosecond pulse propagation in monomode optical fiber and ultrashort pulses. Also, the resulting soliton solutions secured in this study are encouraging and will benefit the community of researchers. In future the investigated model will be solved by some others powerful techniques.

## Data Availability

We have provided all the data within the article.
